# Evaluation of immunomodulatory potential of ethanolic extract of *Roscoea procera rhizomes* in mice

**DOI:** 10.4103/0975-7406.72138

**Published:** 2010

**Authors:** Mahesh S. Sahu, Prashant Y. Mali, Shekhar B. Waikar, Vinod D. Rangari

**Affiliations:** Department of Pharmacognosy, Radharaman Institute of Pharmaceutical Sciences and Radharaman College of Pharmacy, Ratibad, Bhopal-462044, Madhya Pradesh, India; 1 Department of Pharmacology, Radharaman Institute of Pharmaceutical Sciences and Radharaman College of Pharmacy, Ratibad, Bhopal-462044, Madhya Pradesh, India; 2 Department of Pharmacology, Gurunanak College of Pharmacy, Nagpur-440001, Maharashtra, India; 3 Department of Pharmacognosy, J. L. Chaturvedi College of Pharmacy, Nagpur-440016, Maharashtra, India

**Keywords:** Cell-mediated immunity, delayed-type hypersensitivity response, humoral immunity, *Roscoea procera*, rhizomes

## Abstract

**Purpose:**

The aim of present study was to evaluate immunomodulatory potential of ethanolic extract of *Roscoea procera* (Zingiberaceae) rhizomes by using delayed-type hypersensitivity (DTH) and carbon clearance method in comparison to standard established immunosuppressant drug, cyclophosphamide (30 mg/kg, i.p.) in mice.

**Material and Methods:**

The extract was comprised to acute toxicity (OECD-423 guideline), DTH and carbon clearance method for their immunomodulatory potential. Ethanolic extract of *Roscoea procera* rhizomes administered orally at doses 300 mg/kg and 600 mg/kg, p.o. to mice.

**Result and Conclusion:**

Result of our study revealed that, the foot pat thickness of ethanolic extract group (*P*<0.05) significantly enhanced the production of circulating antibody titre in response to Sheep red blood cells (SRBC) and phagocytic functions of mononuclear macrophages and non-specific immunity. Result were also supported by serological and haematological tests data. Hence, the present investigation reveals that, ethanolic extract of *Roscoea procera* rhizomes possesses immunostimulant properties. Further studies to identify the active moieties and elucidation of the mechanism of action are recommended.

Immunomodulators are the substances which modify the activity of the immune system. Immunomodulators have biphasic effects, some tend to stimulate immune system which is low while others inhibit host parameters which are normal or already activated.[1] Immunostimulation and immunosuppression both need to be tackled in order to regulate the normal immunological functioning. Hence, both immunostimulating and immunosuppressing agents have their own standing and search for better agents exerting these activities is becoming the field of major interest all over the world.[2] Immunomodulation using plant material can provide an alternative to conventional chemotherapy for a variety of diseases, especially when the host defense mechanism has to be activated under the condition of impaired immune response.[3] Traditional and folklore medicines play an important role in health services around the globe. About three quarters of the world’s population relies on plants and plant extracts for healthcare. India has an extensive forest cover, enriched with plant diversity. Several plants have been used in folklore medicine.[4] The rational design of novel drugs from traditional medicine offers new prospects in modern healthcare. Ayurveda, the traditional medicinal system in India, describes certain plants whcih strengthen the host immune system.[5] *Roscoea procera* is an ancient Indian medicine, commonly used in several disorders. *Roscoea procera* is one of the essential ingredients of several herbal formulations like tonic and Chyawanprash. The modern screening methods revealed its chemical composition as well as its principle pharmacological activities like spermopiotic, fever, burning, and phthisis, but no attempts have found to substantiate its immunomodulatory activity scientifically.[6] Hence, the present study therefore undertaken to explore the acute toxicity studies and immunomodulatory potential of ethanolic extract of *Roscoea procera* rhizomes on cellular and humoral immune responses to the antigenic challenge by sheep RBCs and by phagocytic activity.

## Materials and Methods

### Plant material

*Roscoea procera* rhizomes were collected from road side clefts of Nainital & Bhavali region, Uttaranchal, India. The plant were authenticated by Dr. N. M. Dongarwar, Department of Botany, R. T. M. University, Nagpur, India, and deposited in the same. (Voucher Specimen No. 2007/9127).

### Preparation of extract

Dried coarsely powdered rhizomes of *Roscoea procera* (400 g) were defatted with petroleum ether (50-60°C) for 72 hrs using Soxhlet apparatus. The marc left subsequently extracted with ethanol (95%, 60-70°C) for 24 hrs. The solvent was removed by distillation under vacuum.[7] The crude brown residue mass of extract were concentrated, stored, and preserved (2-8 °C). The % yield of extract (4.5 w/w) was found on dry wet basis. For dosing, the extract was suspended in 1% Tween-80 to prepare suitable dosage forms.

### Chemicals and reagents

Cyclophosphamide (Khandelwal Laboratories Ltd., Mumbai) and all other solvents used for experimental work were of analytical grade.

### Antigen

Fresh blood was collected from Veterinary College, Nagpur, in sterile Alsevar’s solution. Sheep red blood cells (SRBCs) were washed three times in pyrogen free, sterile saline and centrifuged at 2500-3000 rpm for 10 min. The supernatant was removed with pasture pipette and suspended in normal saline. The concentration of 0.1 ml containing 1×10^8^/mm^3^ cells was adjusted by using improved Neubaur chamber for immunization and challenge.

### Carbon ink suspension

Carbon ink suspension Pelikan-4001, German black ink was injected in a dose of 10 *µ*l/gm body weight of mice.

### Animals

Swiss albino mice (25-30 g) of either sex were used. The animals were fed with standard pellet diet and water *ad libitum* and maintained under standard environmental conditions (22 ± 5°C with 12 h of light/dark cycle). All experimental protocols were approved by Institutional Animal Ethical Committee Clearance (JLCCP/IAEC, 2007/1-10/CPCSEA), J. L. Chaturvedi College of Pharmacy, Nagpur-440016(M.S), India.

### Acute oral toxicity study

Acute oral toxicity studies of extract was carried out as per the OECD guidelines, draft guideline 423 adopted on 17^th^ December, 2001, received from Committee for the Purpose of Control and Supervision of Experiments on Animals (CPCSEA), Ministry of Social Justice and Empowerment, Govt. of India.[8] Administration of stepwise doses of extract of *Roscoea procera* rhizomes from 50 mg/kg body weight up to the dose 5000 mg/kg body weight caused no considerable signs of the toxicity in the tested animals. One tenth of upper limit dose were selected as the level for examination of immunomodulatory potential.

### Delayed type hypersensitivity response

The method described by *Puri* and *Saxena* with some modifications was adopted.[9] Mice were divided into four groups of six mice each. Drugs were administered in various groups, i.e. Group I served as normal control (NC) and received distilled water with Tween 80 (1%), Group II received standard drug cyclophosphamide as an aqueous suspension at a dose of 30 mg/kg, i.p., Group III and IV received extract at a dose of 300 mg/kg and 600 mg/kg body weight as a fine Tween-80 suspension, p.o. All the animals were immunized by injecting 0.1 ml of SRBCs suspension containing 1×10^8^/mm^3^ cells intra-peritoneally on day zero. On day fifth, the thickness of left hind foot pad was measured using Vernier caliper (Mitutoyo Digimatic). The mice were then challenged by injection of 0.1 ml of SRBCs suspension containing 1×10^8^/mm^3^ cells in left hind foot pad. Foot thickness was measured again +24 h, +48 h, + 72 h, and +96 h after this challenge. The blood samples were collected in micro-centrifuge tubes from individual animal by retro-orbital puncture on next day to +96 h and used to analyze WBC and total platelet count. The change in postchallenge foot paw thickness expressed in mm was taken as a measure of DTH reaction. The extract was administered orally on day zero and continued till day ninth (Up to 96 h) before 1 h of measuring foot thickness of challenge. Cyclophosphamide was administered on day zero only.

### Macrophage phagocytosis by carbon clearance method

The method described by *Hudson* and *Hay* was followed with some modifications.[10] Mice were divided into four groups of six mice each. Drugs were administered in various groups, i.e. Group I served as normal control (NC) and received distilled water with Tween-80 (1%), Group II received standard drug cyclophosphamide as an aqueous suspension at a dose of 30 mg/kg, i. p., Group III and IV received extract at a dose of 300 mg/kg and 600 mg/kg body weight, as a fine Tween-80 suspension, p.o., for fifth day prior to injection of carbon particles. On day six, all the groups were given 0.1 ml of carbon ink suspension through the tail vein. Blood was collected from the retro-orbital plexuses of individual animals at 0 and 15 min immediately after the injection of carbon suspension. Blood (25 *µ*l) was lysed with 2 ml of 0.1% sodium carbonate and the absorbance was measured spectrophotometrically at 675 nm for determination of optical densities. The rate of carbon clearance, termed as phagocytic index (K), was calculated by using equation.[11]

$$K=\;(\mathrm{In}\;{\mathrm{OD}}_{1}\;-\;\mathrm{In}\;{\mathrm{OD}}_{2})/t_{2}-t_{1}$$

Where OD_1_ Optical density at a time (*t*_1_) 0 min after blood collection from mice tail vein

(Optical density is the absorbance of an optical element for a given wavelength).

OD_2_ Optical density at a time (*t*_2_) 15 min after blood collection from mice tail vein.

### Statistical analysis

Data were expressed as a Mean ± S.E.M., using one way analysis of variance (ANOVA) followed by Dunnett’s *t*-test. *P*< 0.05 was considered as statistical significant, *n* = 6 in each group.

## Results and Discussion

DTH is a part of the process of graft rejection, tumor immunity, and most importantly immunity to many intracellular infectious microorganisms especially those causing chronic diseases such as tuberculosis.[12] DTH requires the specific recognition of a given antigen by activated T lymphocytes, which subsequently proliferate and release cytokines. These in turn increase vascular permeability, induce vasodilatation, macrophage accumulation and activation, promoting increased phagocytic activity and increased concentrations of lytic enzymes for more effective killing.[13,14] The effect of ethanolic extract of *Roscoea procera* rhizomes on foot pad thickness and hematological data such as WBC and total platelet counts of antigenically challenged mice in [Tables 1 and 2] showed, there was (*P*< 0.05) significant decrease in foot pad thickness, WBC and total platelet counts in cyclophosphamide group as compared to control group. While in extract-treated group animals showed (*P*< 0.05) significant increase. Control of disease by immunological means has two aspects, namely the development and improvement of protective immunity and avoidance of undesired immunological side reactions. Modulation of the immune system by cytostatic agents is emerging as a major area in pharmacology, especially in cases, where undesired immunosuppression is the result of therapy. Cytotoxic drugs like cyclophosphamide and azathioprin act at various levels on cells involved in defence against foreign invaders.[15] Immunomodulatory agents can enhance or inhibit the immunological responsiveness of an organism by interfering with its regulatory mechanisms. They may selectively activate either cell-mediated or humoral immunity by stimulating either TH1 or TH2 type cell response, respectively. Immunomodulatory agents that are free from side effects and which can be administered for long duration to obtain a continuous immune activation are highly desirable for the prevention of diseases. There are a variety of naturally and chemically derived compounds discovered with immunomodulatory activity such as levamosole, glucan, IL-2, interferon, etc., which are used in combination with cisplatin, adiramycin, 5-flurouracil, etc. against many types of carcinomas. But most of these immunomodulatory compounds have side effects namely fever, myaligias fatigue, etc.[16] The role of phagocytosis is primarily the removal of microorganisms and foreign bodies, but also the elimination of dead or injured cells. Phagocytic defects are associated with varied pathological conditions in humans.[17] In view of the pivotal role played by the macrophages in coordinating the processing and presentation of antigen to β-cells. Phagocytic index [Table 3] were (*P*<0.05) significantly increased as compared to control and cyclophosphamide group. Hence, increased clearance rate of carbon particles from circulation in animal reflects the enhancement of phagocytic function of mononuclear macrophage and non-specific immunity. Phagocytosis by macrophages is important against pathogenic microorganisms and its effectiveness is markedly enhanced by opsonisation of parasite with antibodies and complement C3b leading to more rapid clearance of parasite from blood.[18] The modulation of immune response using medicinal plant products as a possible therapeutic measure has become a subject of active scientific investigations.[19] Based on our results, ethanolic extract of *Roscoea procera* rhizomes was found to have a promising immunostimulant potential. It may be due to increase in DTH reaction in mice in response to T-cell dependent antigen stimulatory effect on lymphocytes and necessary cell types required for the expression of reaction. DTH response is direct co-related to cell-mediated immunity and was significantly increased with ethanolic extract of *Roscoea procera* rhizomes as compared to normal control. The significant increase in immunostimulatory potential of ethanolic extract of *Roscoea procera* rhizomes could be attributed due to presence of flavonoids, alkaloids, tannins, saponin glycosides, phenolic compounds, etc. Further studies to identify the active moieties and elucidation of the exact mechanism of action are recommended. Thus, present study validates the traditional use of *Roscoea procera* rhizomes in Ayurvedic system of medicine.

**Table 1:** Effect of ethanolic extract of *Roscoea procera* rhizomes on foot pad thickness of antigenically (SRBCs suspension) challenged mice

Groups	Mean foot pad thickness (mm)			
	24 hrs	48 hrs	72 hrs	96 hrs
Control (Tween-80, p.o.)	0.51±0.07	0.37±0.06	0.28±0.05	0.19±0.03
Cyclophosphamide (30 mg/kg, i.p.)	0.46±0.06	0.31±0.06*	0.23±0.04*	0.14±0.04**
EERPR (300 mg/kg, p.o.)	0.62±0.05	0.48±0.06*	0.40±0.02**	0.33±0.01**
EERPR (600 mg/kg, p.o.)	0.67±0.09	0.54±0.04*	0.45±0.05**	0.39±0.03**

Data were expressed as a Mean ± S.E.M, using one way analysis of variance (ANOVA) followed by Dunnett’s *t*-test. *P*<0.05 was considered as statistical significant, *n* = 6 in each group. EERPR- Ethanolic extract of *Roscoea procera* rhizomes

**Table 2:** Effect of ethanolic extract of *Roscoea procera* rhizomes on WBC count and total platelet count of antigenically (SRBCs suspension) challenged mice

Groups	WBC count (1000/mm^3^)	Total platelet count (1000/mm^3^)
Control (Tween-80, p.o.)	5.45±2.62	469.3±246.5
Cyclophosphamide (30 mg/kg, i.p.)	4.51±1.70*	401.5±216.1*
EERPR (300 mg/kg, p.o.)	6.03±2.41**	574.4±337.6*
EERPR (600 mg/kg, p.o.)	7.81±2.51**	677.5±226.1**

Data were expressed as a Mean ± S.E.M, using one way analysis of variance (ANOVA) followed by Dunnett’s *t*-test, *P*<0.05 was considered as statistical significant, *n* = 6 in each group. EERPR- Ethanolic extract of *Roscoea procera* rhizomes

**Table 3:** Effect of ethanolic extract of *Roscoea procera* rhizomes on phagocytic index by using carbon clearance method

Groups	Phagocytic Index
Control (Tween-80, p.o.)	0.0111±0.0036
Cyclophosphamide (30 mg/kg, i.p.)	0.0085±0.0026*
EERPR (300 mg/kg, p.o.)	0.0145±0.0026**
EERPR (600 mg/kg, p.o.)	0.0187±0.0047**

Data were expressed as a Mean ± S.E.M, using one way analysis of variance (ANOVA) followed by Dunnett’s *t*-test. *P*<0.05 was considered as statistical significant, *n* = 6 in each group. EERPR- Ethanolic extract of *Roscoea procera* rhizomes
